# Risk Factors and Cumulative Incidence of Cystoid Macular Edema After Simple Cataract Surgery: A Systematic Review and Meta-Analysis

**DOI:** 10.7759/cureus.84212

**Published:** 2025-05-16

**Authors:** Rafaela Smarlamaki, Eleni-Andromachi Koutsoumpi, Theodora Psaltopoulou, Georgios Kalarakis, Panagiotis Theodossiadis, Ilias Georgalas, Vasileios Peponis, Irini Chatziralli, Theodoros N Sergentanis

**Affiliations:** 1 Department of Ophthalmology, Ophthalmiatreio Athens Hospital, Athens, GRC; 2 Department of Ophthalmology, National and Kapodistrian University of Athens School of Medicine, Athens, GRC; 3 Department of Clinical Therapeutics, “Alexandra” Hospital, School of Medicine, Athens, GRC; 4 Department of Neuroradiology, Karolinska University Hospital, Stockholm, SWE; 5 2nd Department of Ophthalmology, National and Kapodistrian University of Athens School of Medicine, Athens, GRC; 6 Department of Medicine and Surgery, National and Kapodistrian University of Athens School of Medicine, Athens, GRC

**Keywords:** cataract, cystoid macular edema, irvine-gass, meta-analysis, phacoemulsification, post-operative macular edema, pseudophakic

## Abstract

Pseudophakic cystoid macular edema (PCME) is a common side effect following cataract surgery, especially in patients with systemic and/or ocular comorbidities. Research evidence has established the impact of cataract surgery on PCME occurrence; various risk factors could contribute to PCME development, as indicated by epidemiological studies. This research aims to examine the cumulative incidence of PCME and explore the correlation between PCME occurrence, ophthalmic, systemic, and surgery-related risk factors after uncomplicated cataract surgery.

PubMed and EMBASE databases were used for this systematic search (end-date of search: March 31, 2024). Random-effects (DerSimonian-Laird) models were used to estimate the pooled cumulative incidence, pooled relative risk (RR), and 95% confidence intervals (95% CIs). Subgroup analyses, sensitivity analyses, and meta-regression analyses were also carried out.

The meta-analysis concerning cumulative PCME incidence included 143 studies. Thirty-seven studies were included in a meta-analysis of possible PCME risk factors. Pooled cumulative PCME incidence was found to be 5%. PCME was more frequent in patients with diabetes mellitus (DM) (RR=2.87, 95%Cl: 1.96-4.21), epiretinal membrane (ERM) (RR=4.51, 95%Cl: 3.06-6.64), uveitis (RR=6.76, 95%Cl: 2.49-18.31), pars plana vitrectomy (PPV) (RR=4.11, 95%Cl: 1.86-9.11) and retinal vein occlusion (RVO) (RR=8.79, 95%Cl: 1.90-40.76).

DM, ERM, uveitis, PPV, and RVO were associated with PCME. Additional prospective cohort studies investigating various risk factors seem desirable.

## Introduction and background

Pseudophakic cystoid macular edema (PCME), widely known as “Irvine-Gass syndrome,” is considered the primary cause of visual impairment following cataract surgery, remaining the most common postoperative complication [1-7]. The reported incidence of clinical CME after cataract surgery has ranged from 0.1% to 2.35% [4], although in a recent database study on 81,984 eyes, the incidence of CME was reported to be 1.17%-4.04% [5]. A significant decrease in the incidence of pseudophakic CME has been reported due to advances in cataract surgery with the introduction of small-incision phacoemulsification, which results in reduced blood-aqueous barrier (BAB) damage compared to previously used intra- or extra-capsular cataract extraction [8,9].

The pathogenesis of PCME remains unclear and is considered multifactorial. The type of cataract surgery, light toxicity, vitreomacular traction, inflammatory mediators, use of adrenergic drugs, age, vitreous loss, integrity of the posterior capsule, hypertension, and diabetes are considered contributing factors to CME development [10,11]. Inflammation, which is induced due to surgical maneuvers, is a major cause of PCME, since inflammatory mediators lead to breakdown of the blood-aqueous and blood-retinal barriers (BRBs) and increase vascular permeability, resulting in cyst formation especially in the outer plexiform and inner nuclear retinal layers [12,13], although lamellar holes and subretinal fluid presence have been also described [14].

PCME is categorized as angiographic or subclinical CME when it is observed as leakage on fluorescein angiography in otherwise asymptomatic patients, or as clinical when it is accompanied by visual acuity impairment, usually around four to six weeks after cataract surgery [13]. Of note, angiographic CME detection is much more frequent, with varying incidence, up to 70% in some studies [13]. Apart from the characteristic fluorescein angiography “petalloid” pattern in the late phase and the staining of the optic nerve, which may also occur, contributing to the differential diagnosis of PCME from other CME causes, optical coherence tomography (OCT) confirms the diagnosis of CME. Specifically, in OCT, PCME is presented with loss of the foveal depression, retinal thickening, and cystic hyporeflective lesions, while OCT also allows the detection of vitreoretinal traction and lamellar holes [14]. In terms of duration, CME can be classified as acute when it occurs within six months or chronic when it happens over six months postoperatively [13].

An increase in PCME risk has been associated with multiple ophthalmic, systemic, and surgery-related conditions, with diabetes being the most common [15-17]. Based on the above, the purpose of this research was to evaluate PCME's cumulative incidence, as well as possible systemic (diabetes mellitus (DM), hypertension), ocular (diabetic retinopathy (DR), uveitis, retinal vein occlusion (RVO), posterior vitreous detachment (PVD), age-related macular degeneration (AMD)), and surgery-related (pars-plane vitrectomy, femtosecond laser-assisted cataract surgery (FLACS), small-incision cataract surgery (SICS)) risk factors for its development.

## Review

Material and methods

Search Strategy and Eligibility of Studies

The present systematic review and meta-analysis followed the Preferred Reporting Items for Systematic Reviews and Meta-Analyses (PRISMA) guidelines. All authors discussed and agreed upon the study protocol in advance. PubMed and EMBASE databases were used for this systematic search (end-date of search: March 31, 2024). The search method included the following algorithm: (pseudophakic OR postoperative OR cystoid OR Irvine-Gass) AND (macular OR macula) AND (edema OR oedema) AND (phacoemulsification OR cataract). A restriction in English manuscripts was implemented regarding the publication language. A “snowball” procedure was used for relevant articles via a search of reference lists of reviews and eligible articles.

Eligible articles included randomized controlled trials, cohort, case-control studies, and case series (≥20 eyes) investigating the association between uncomplicated cataract surgery and PCME. Case series including less than 20 patients and case reports, reviews, were not included in this meta-analysis. Only the larger study was inserted in case of overlapping study populations. Two reviewers (RS, EK) working independently performed the selection of studies and consultation with two senior authors (IC, TNS), and team consensus was carried out in case of any disagreements.

Data Abstraction and Effect Estimates

General information (first author’s name, study year), study characteristics (study design, time period, geographical region, cohort size and incidence cases (for cohort studies), number of cases and controls (for case-control studies)), matching factors, maximum follow-up period of patients, characteristics of participants (inclusion and exclusion criteria, age of participants (range, mean), percentage of males, ethnicity, inclusion and exclusion criteria, systemic comorbidities, ophthalmic comorbidities, initial mean central subfield macular thickness (CSMT), initial mean best-corrected visual acuity (BCVA)), definition of PCME, categorization of exposure, ascertainment of risk factors, and any adjusting factors concerning the multivariate analyses were used in the data abstraction. The corresponding authors were contacted twice (a reminder e-mail was sent seven days after the first e-mail), in case the required data for the meta-analysis were not readily available in the published article. Two reviewers (RS and EK) extracted the data, analyzed it, and recorded it in a predeveloped data extraction form, independently. Consultation with two senior authors (TNS and IC) and team consensus was performed, and the final decision was reached.

The necessary data for calculation of PCME cumulative incidence, as well as maximally adjusted effect estimates, i.e., odds ratios (ORs), relative risks (RRs) or hazard ratios (HRs) with their 95% confidence intervals (CIs), were extracted from each study by category of exposure of CME development. All effect estimates were appropriately transformed into RRs. In case the aforementioned information was not available, crude effect estimates and 95% CIs were calculated using 2x2 tables presented in the articles.

Statistical Analyses

Random-effects (DerSimonian-Laird) models were appropriately used to calculate pooled effect estimates. In the analysis of cumulative incidence rates, the arcsine (Freeman-Tukey) transformation was implemented. In case of two or more eligible study arms, a statistical synthesis was performed. The category of patients/eyes with CME or probable risk factor was compared with the one without CME/risk factor. The presence of DM, hypertension, DR, epiretinal membrane (ERΜ), uveitis, pars plana vitrectomy (PPV), RVO, AMD, dry AMD, PVD, femtosecond laser-assisted cataract surgery (FLACS), and small-incision cataract surgery (SICS) were examined as risk factors. Estimation of I^2^ was used to assess between-study heterogeneity.

Subgroup analyses in regard to study design, geographic region (grouped as European, Asian, North and South American, and Australian), eye- or patient-based analysis, and overall study quality were carried out. Meta-regression analysis was performed in order to assess whether PCME cumulative incidence was associated with gender (indicated as the percentage of males in the individual studies), age (indicated as the mean age in the individual studies), and year of publication. STATA/SE version 13.1 (Stata Corp., College Station, TX, USA) was used to perform Statistical analysis and meta-regression analysis.

Assessment of Study Quality and Publication Bias

The Newcastle-Ottawa Quality scale was implemented to evaluate the quality of the included studies to evaluate the risk of bias. The cut-off value was set a priori at more than or equal to three months follow-up and ≥85% follow-up rate, respectively, regarding the items assessing the completeness (adequacy) of follow-up of cohorts and whether the follow-up period was enough for outcomes to occur. The maximum score of the Newcastle-Ottawa scale was modified in the incidence study. In particular, the items “Selection of unexposed” and “Comparability on age and other factors” were assessed as N/A (not applicable). This way, the maximum score was modified to 6.

The rating of studies was performed by two independently working reviewers (RS, EK) and, in case of disagreement, the final decision was reached after consultation with a senior author (TΝS) and team consensus. Publication bias was assessed in the analyses including 10 or more study arms; Egger’s statistical test was carried out, and a visual inspection of the funnel plot was conducted. Statistical significance was defined as p<0.1 in order to interpret the Egger’s test [18]. STATA/SE version 13.1 was used to evaluate the publication bias. 

Results

Description of Eligible Studies

**Figure 1:**
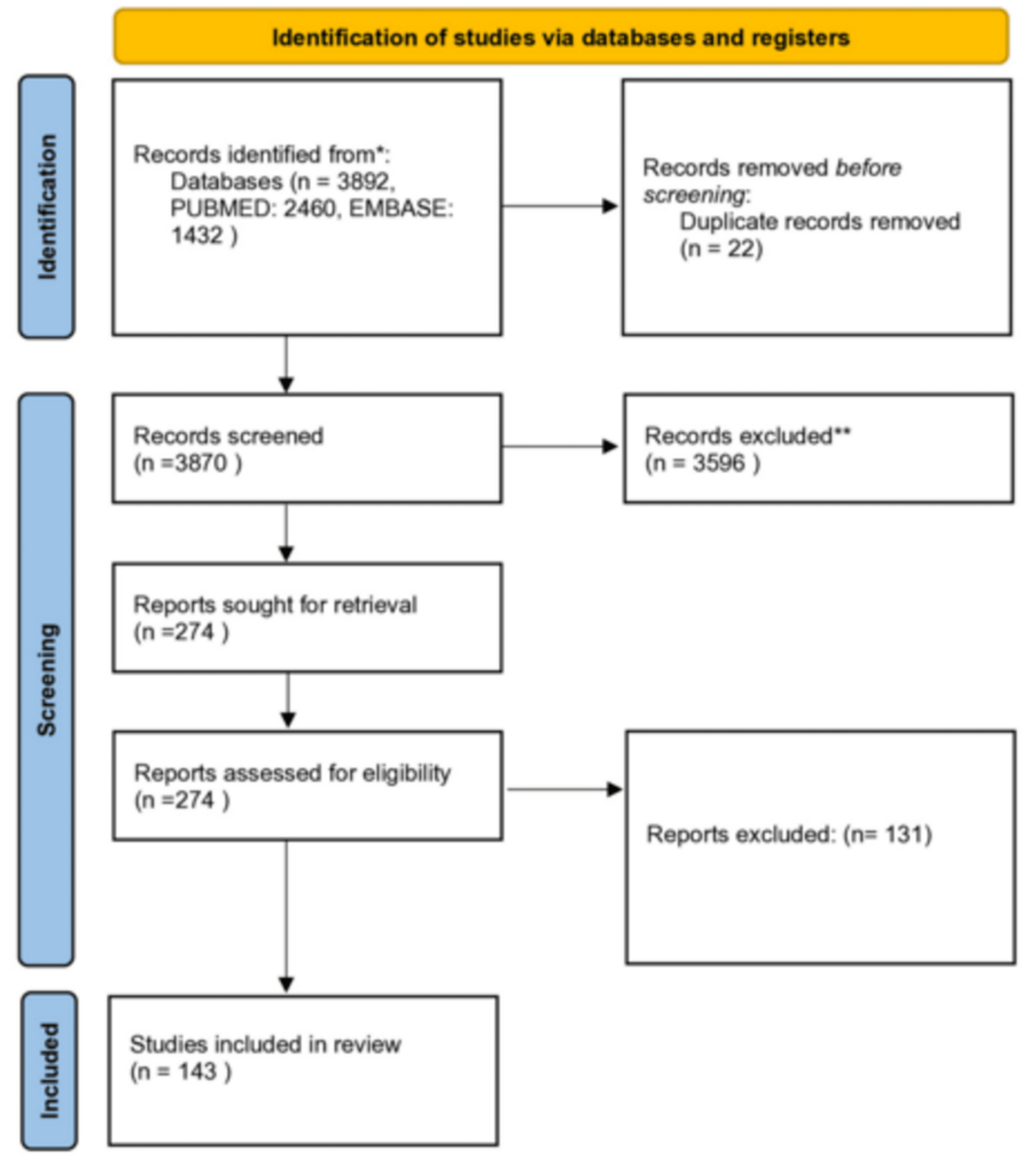
PRISMA flow chart presenting the successive steps in the selection of eligible studies. n: number of studies, PRISMA: Preferred Reporting Items for Systematic Reviews and Meta-Analyses *all studies presented univariate estimates and therefore no subgroup analysis by adjustment was presented; **all studies presented multivariate estimates and therefore no subgroup analysis by adjustment was presented

The algorithm led to an identification of 3,892 records, which were performed (2,460 PubMed-derived, 1,432 EMBASE-derived using the filter of non-MEDLINE items). About 3,870 titles and abstracts were screened after the removal of duplicates (Figure 1).

One hundred forty-three studies exploring the association between cataract surgery and PCMEs’ development cumulative incidence, were finally considered eligible [4,5,11,19-160]. In 16 of these studies, only conference abstracts were available. All studies were cohort, except for one study [75], which was a cohort in cumulative incidence assessment and a case-control study concerning the risk factor evaluation. The studies by Scheers et al. [23,40] had overlapping data concerning cumulative incidence. As a result, the more recent one was selected for the analysis.

Meta-Analysis

The estimation of pooled cumulative incidence of PCME was found to be 5% (n=143 articles; pooled effect size (ES)=5%, 95%CI: 5%-6%) (Tables 1, 2, Figure 1). This pattern was similar in the subgroup analysis by publication year for studies conducted during the last decade (pooled ES=5%, 95% Cl: 4%-5%), in eye-based (pooled ES=5%, 95% CI: 4%-5%) and patient-based (pooled ES=5%, 95% CI: 3%-7%) subgroup analyses.

**Table 1:** Description of the association between uncomplicated cataract surgery and cumulative incidence of cystoid macular edema. ES: effect size; 95%CI: 95 percent confidence interval; I^2^: evolution of heterogeneity; p: p-value Presentation of sub-analyses per publication year [4,5,11,19-160].

Study	ES (95% Cl)	% Weight
2014 or later		
Alsarhani et al. (2022)	0.03 (0.02, 0.06)	0.90
Anastasilakis et al. (2015)	0.04 (0.01, 0.09)	0.67
Antonio-Aguirre et al. (2022)	0.01 (0.01, 0.01)	1.13
Ashraf et al. (2018)	0.22 (0.09, 0.45)	0.23
Assil et al. (2015)	0.00 (0.00, 0.07)	0.48
Bacquet et al. (2015)	0.25 (0.13, 0.43)	0.32
Bajraktari et al. (2023)	0.17 (0.11, 0.25)	0.67
Bamahfouz (2021)	0.22 (0.16, 0.30)	0.74
Banker et al. (2014)	0.22 (0.15, 0.32)	0.60
Bellur et al. (2022)	0.06 (0.05, 0.06)	1.13
Bezkorovayna et al. (2020)	0.16 (0.10, 0.25)	0.60
Bhargava et al. (2022)	0.13 (0.10, 0.18)	0.90
Cai et al. (2015)	0.05 (0.04, 0.06)	1.05
Chaudhary et al. (2015)	0.01 (0.01, 0.04)	0.83
Chiras et al. (2021)	0.02 (0.01, 0.03)	1.03
Chiu et al. (2017)	0.09 (0.06, 0.16)	0.74
Cho et al. (2018)	0.27 (0.20, 0.36)	0.69
Chu et al. (2016)	0.02 (0.01, 0.02)	1.13
Cioana et al. (2024)	0.20 (0.14, 0.27)	0.76
Conrad−Hengerer et al. (2014)	0.02 (0.01, 0.06)	0.84
Copete et al. (2019)	0.12 (0.07, 0.19)	0.69
Crymes et al. (2019)	0.02 (0.01, 0.03)	1.03
Dabas et al. (2022)	0.00 (0.00, 0.03)	0.70
Daien et al. (2016)	0.01 (0.01, 0.01)	1.12
Denier et al. (2018)	0.08 (0.03, 0.18)	0.48
Do et al. (2015)	0.11 (0.07, 0.17)	0.76
Doncel-Fernandez et al. (2021)	0.00 (0.00, 0.02)	0.80
Dong et al. (2015)	0.29 (0.22, 0.38)	0.70
Du et al. (2023)	0.18 (0.11, 0.27)	0.64
Dvali et al. (2019)	0.00 (0.00, 0.07)	0.45
Folden & Wong (2022)	0.04 (0.01, 0.13)	0.48
Ganesh et al. (2017)	0.00 (0.00, 0.00)	1.10
Garcia Gomez de Segura et al. (2022)	0.09 (0.04, 0.17)	0.56
Gehlsen et al. (2022)	0.04 (0.02, 0.09)	0.67
Golebiewska et al. (2014)	0.00 (0.00, 0.05)	0.58
Gudauskiene et al. (2020)	0.03 (0.01, 0.11)	0.53
Haleem et al. (2017)	0.02 (0.01, 0.08)	0.61
Hanna et al. (2014)	0.08 (0.03, 0.20)	0.40
Hardin et al. (2018)	0.01 (0.01, 0.01)	1.13
Hengerer et al. (2016)	0.02 (0.01, 0.05)	0.85
Ivastinovic et al. (2017)	0.13 (0.06, 0.26)	0.44
Jaafar et al. (2022)	0.12 (0.06, 0.20)	0.61
Javidi & Mahmood (2016)	0.02 (0.01, 0.02)	1.10
Jeong et al. (2023)	0.55 (0.45, 0.65)	0.62
Jiang et al. (2018)	0.01 (0.00, 0.05)	0.66
Karimi et al. (2019)	0.00 (0.00, 0.03)	0.67
Kemer Atik et al. (2021)	0.00 (0.00, 0.04)	0.62
Khan et al. (2020)	0.06 (0.04, 0.11)	0.77
Khodeiry et al. (2021)	0.00 (0.00, 0.11)	0.33
Kishan et al. (2024)	0.05 (0.03, 0.08)	0.92
Kocak Altintas et al. (2016)	0.07 (0.03, 0.13)	0.70
Kochhar et al. (2014)	0.00 (0.00, 0.04)	0.66
Kumar et al. (2022)	0.04 (0.02, 0.07)	0.83
Lee et al. (2017)	0.33 (0.25, 0.41)	0.74
Lehmann et al. (2021)	0.01 (0.00, 0.03)	0.93
Levitz et al. (2015)	0.01 (0.01, 0.02)	1.07
Lin et al. (2014)	0.18 (0.12, 0.27)	0.67
Lin et al. (2021)	0.07 (0.06, 0.09)	1.09
Lindsey et al. (2015)	0.02 (0.02, 0.03)	1.11
List et al. (2022)	0.01 (0.01, 0.01)	1.13
Ma et al. (2021)	0.00 (0.00, 0.04)	0.63
Magone et al. (2019)	0.01 (0.01, 0.02)	1.07
Menon et al. (2023)	0.07 (0.02, 0.22)	0.33
Miller et al. (2021)	0.02 (0.02, 0.02)	1.12
Mirachtsis et al. (2016)	0.11 (0.08, 0.16)	0.85
Moreira Neto et al. (2015)	0.00 (0.00, 0.04)	0.65
Nithianandan et al. (2019)	0.01 (0.00, 0.01)	1.10
Oyewole et al. (2017)	0.08 (0.05, 0.11)	0.88
Ozates et al. (2020)	0.18 (0.11, 0.26)	0.65
Ozturk et al. (2016)	0.05 (0.02, 0.10)	0.68
Padidam et al. (2022)	0.09 (0.06, 0.13)	0.87
Palkar et al. (2020)	0.05 (0.01, 0.17)	0.40
Pierru et al. (2014)	0.03 (0.01, 0.07)	0.69
Saboo et al. (2015)	0.03 (0.01, 0.11)	0.52
Samanta et al. (2014)	0.39 (0.30, 0.49)	0.64
Schallhorn et al. (2023)	0.04 (0.02, 0.08)	0.82
Schaub et al. (2016)	0.21 (0.16, 0.26)	0.87
Scheers et al. (2023)	0.01 (0.01, 0.02)	1.07
Schwarzenbacher (2019)	0.00 (0.00, 0.04)	0.63
Schwarzenbacher et al. (2024)	0.00 (0.00, 0.03)	0.70
Seth et al. (2022)	0.11 (0.06, 0.19)	0.61
Sever & Horozoğlu (2018)	0.01 (0.01, 0.02)	1.07
Singh et al. (2019)	0.03 (0.01, 0.17)	0.33
Singh et al. (2019)	0.00 (0.00, 0.09)	0.40
Sotiropulos et al. (2023)	0.32 (0.23, 0.42)	0.60
Takeda et al. (2021)	0.02 (0.00, 0.08)	0.54
Van Nuffel et al. (2020)	0.03 (0.01, 0.06)	0.88
Walsh et al. (2016)	0.09 (0.07, 0.11)	1.04
Xia et al. (2022)	0.03 (0.02, 0.04)	1.09
Xu et al. (2019)	0.02 (0.01, 0.02)	1.09
Yang et al. (2017)	0.05 (0.04, 0.06)	1.05
Yao et al. (2023)	0.00 (0.00, 0.08)	0.43
Ylinen et al. (2017)	0.03 (0.01, 0.09)	0.64
Zhang et al. (2017)	0.14 (0.08, 0.23)	0.57
de Melo Franco et al. (2015)	0.06 (0.02, 0.13)	0.56
Subtotal (I^2^ = 98.57%, p = 0.00)	0.05 (0.04, 0.05)	71.45
Before 2014		
Abdelmoaty et al. (2006)	0.01 (0.00, 0.03)	0.80
Barsam et al. (2008)	0.00 (0.00, 0.06)	0.53
Berker et al. (2004)	0.14 (0.06, 0.28)	0.38
Biro et al. (2008)	0.00 (0.00, 0.05)	0.56
Cagini et al. (2009)	0.03 (0.01, 0.11)	0.52
Ching et al. (2006)	0.03 (0.01, 0.08)	0.73
Chu et al. (2013)	0.08 (0.05, 0.12)	0.90
Colin (1996)	0.00 (0.00, 0.04)	0.64
Davison (2002)	0.01 (0.00, 0.01)	1.10
Degenring et al. (2007)	0.33 (0.25, 0.42)	0.70
Dholakia & Vasavada (2004)	0.00 (0.00, 0.02)	0.78
Dowler et al. (2000)	0.30 (0.19, 0.45)	0.44
Ecsedy et al. (2011)	0.00 (0.00, 0.09)	0.40
Eriksson et al. (2011)	0.09 (0.04, 0.18)	0.55
Estafanous et al. (2001)	0.33 (0.21, 0.49)	0.40
Gharbiya et al. (2013)	0.05 (0.01, 0.16)	0.42
Ghosh et al. (2010)	0.00 (0.00, 0.02)	0.82
Gulkilik et al. (2006)	0.26 (0.18, 0.35)	0.65
Hayashi et al. (2009)	0.13 (0.07, 0.23)	0.55
Horozoglu et al. (2011)	0.00 (0.00, 0.15)	0.27
Jain et al. (2001)	0.02 (0.01, 0.05)	0.88
Javadi et al. (2005)	0.02 (0.00, 0.13)	0.41
Katsimpris et al. (2012)	0.16 (0.10, 0.25)	0.65
Kawaguchi et al. (2007)	0.06 (0.03, 0.12)	0.73
Kim et al. (2007)	0.22 (0.13, 0.36)	0.46
Kim (2008)	0.14 (0.09, 0.21)	0.72
Kosker et al. (2013)	0.02 (0.00, 0.10)	0.49
Kurz et al. (2009)	0.00 (0.00, 0.05)	0.56
Kusbeci et al. (2012)	0.13 (0.08, 0.22)	0.63
Kwon et al. (2011)	0.18 (0.12, 0.27)	0.67
Lobo et al. (2004)	0.28 (0.16, 0.45)	0.35
Masood et al. (2007)	0.35 (0.22, 0.50)	0.40
Mentes et al. (2003)	0.09 (0.06, 0.13)	0.88
Monden (2002)	0.09 (0.05, 0.15)	0.67
Mylonas et al. (2013)	0.14 (0.06, 0.29)	0.37
Nagy et al. (2012)	0.00 (0.00, 0.13)	0.29
Perente et al. (2007)	0.29 (0.21, 0.38)	0.68
Rauz et al. (2000)	0.09 (0.04, 0.20)	0.49
Romero−Aroca et al. (2006)	0.02 (0.00, 0.05)	0.73
Sahin et al. (2013)	0.08 (0.03, 0.20)	0.40
Sarda et al. (2012)	0.08 (0.03, 0.20)	0.45
Shingleton et al. (2006)	0.00 (0.00, 0.02)	0.87
Squirrell et al. (2002)	0.05 (0.01, 0.15)	0.43
Stifter et al. (2008)	0.00 (0.00, 0.04)	0.66
Ursell et al. (1999)	0.19 (0.13, 0.28)	0.66
Vukicevic et al. (2012)	0.13 (0.06, 0.27)	0.40
Yuksel et al. (2008)	0.03 (0.01, 0.10)	0.62
Zaczek & Zetterström (1999)	0.11 (0.06, 0.20)	0.57
von Jagow et al. (2007)	0.00 (0.00, 0.11)	0.34
Subtotal (I^2^ = 93.49%, p = 0.00)	0.07 (0.04, 0.10)	28.55
Heterogeneity between groups: p = 0.059		
Overall (I^2^ = 98.10%, p = 0.00);	0.05 (0.05, 0.06)	100.00

**Table 2:** Results of the meta-analyses examining the pooled cumulative incidence of PCME. §number of studies; ES: effect estimate, I^2^: evolution of heterogeneity; %: percent; PCME: Pseudophakic cystoid macular edema Subgroup analyses per study design and geographic region are presented.

	n^§^	ES (95%CI)	Heterogeneity I^2^	P-value
Analysis of cumulative incidence				
Overall analysis	143	0.05 (0.05-0.06)	98.11%	<0.001
Subgroups by publication year				
2014 or later	94	0.05 (0.04-0.05)	98.59%	<0.001
Before 2014	49	0.07 (0.04-0.10)	93.49%	<0.001
Subgroups by study design				
Eye-based analysis	124	0.05 (0.04-0.05)	96.33%	<0.001
Patient-based analysis	19	0.05 (0.03-0.07)	98.35%	<0.001
Subgroups by geographic region				
Asia	58	0.07 (0.05-0.09)	95.51%	<0.001
Australia	4	0.03 (0.01-0.06)	89.29%	<0.001
Europe	55	0.04 (0.04-0.05)	95.14%	<0.001
North America	25	0.05 (0.03-0.06)	99.46%	<0.001
South America	1	0.00 (0.00-0.04)	NC	

Thirty-seven studies were included in the meta-analysis examining associations between risk factors and PCME development.

**Figure 2:**
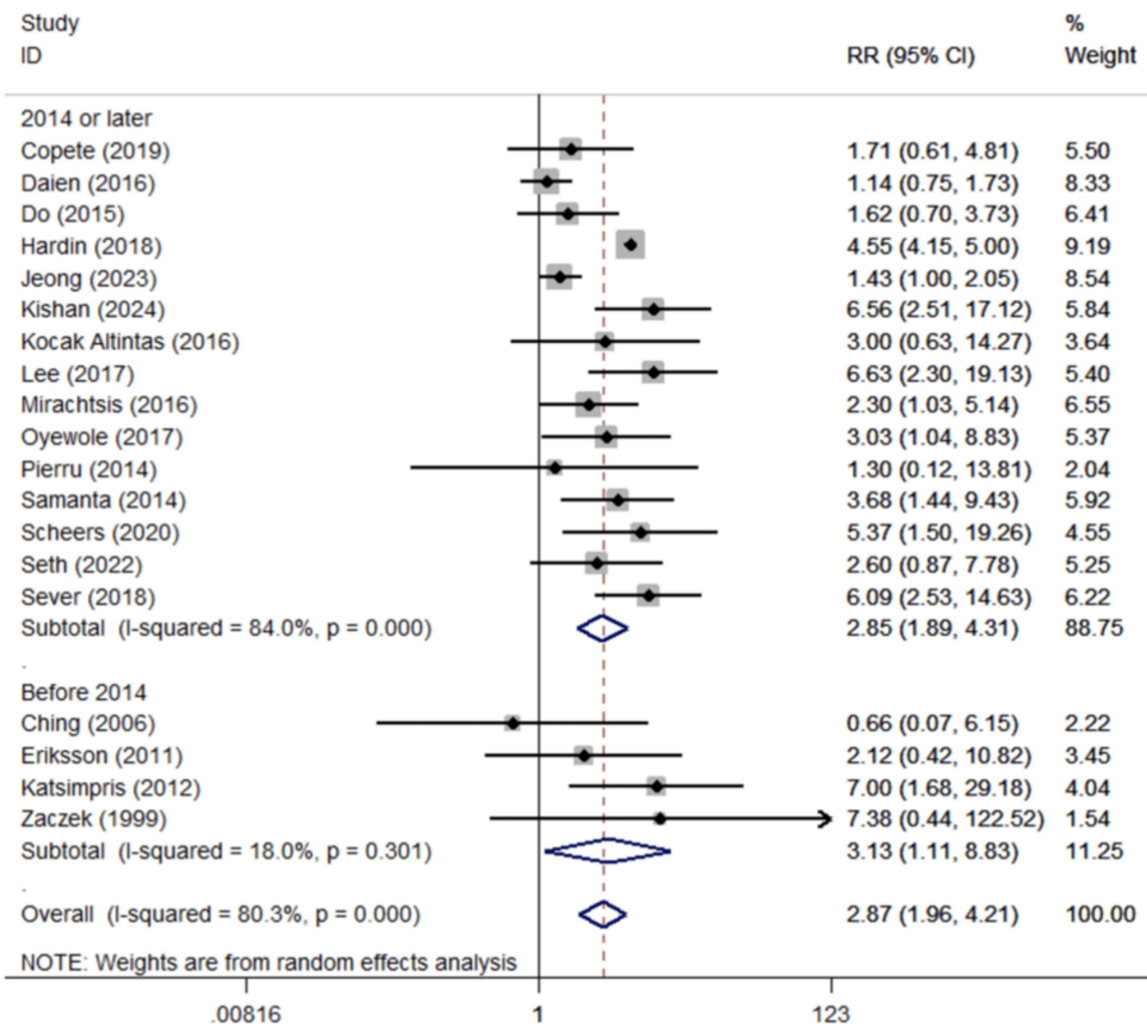
Forest plot describing the association between diabetes mellitus and CME occurrence following uncomplicated cataract surgery. ID: identification; RR: relative risk; %: percent; p: p-value; I-squared: used for heterogeneity estimation; CME: cystoid macular edema Presentation of sub-analyses per publication year. Bold cells denote statistically significant associations.

Diabetes mellitus: Nineteen studies examined the association between DM and risk of PCME (Figure 2) [20-38], with their synthesis showing a statistically significant association (pooled RR=2.87, 95%Cl: 1.96-4.21). The significant associations persisted at the subgroup analyses by level of publication year (Figure 2), adjustment, and geographical region, for Asian and European populations.

**Figure 3:**
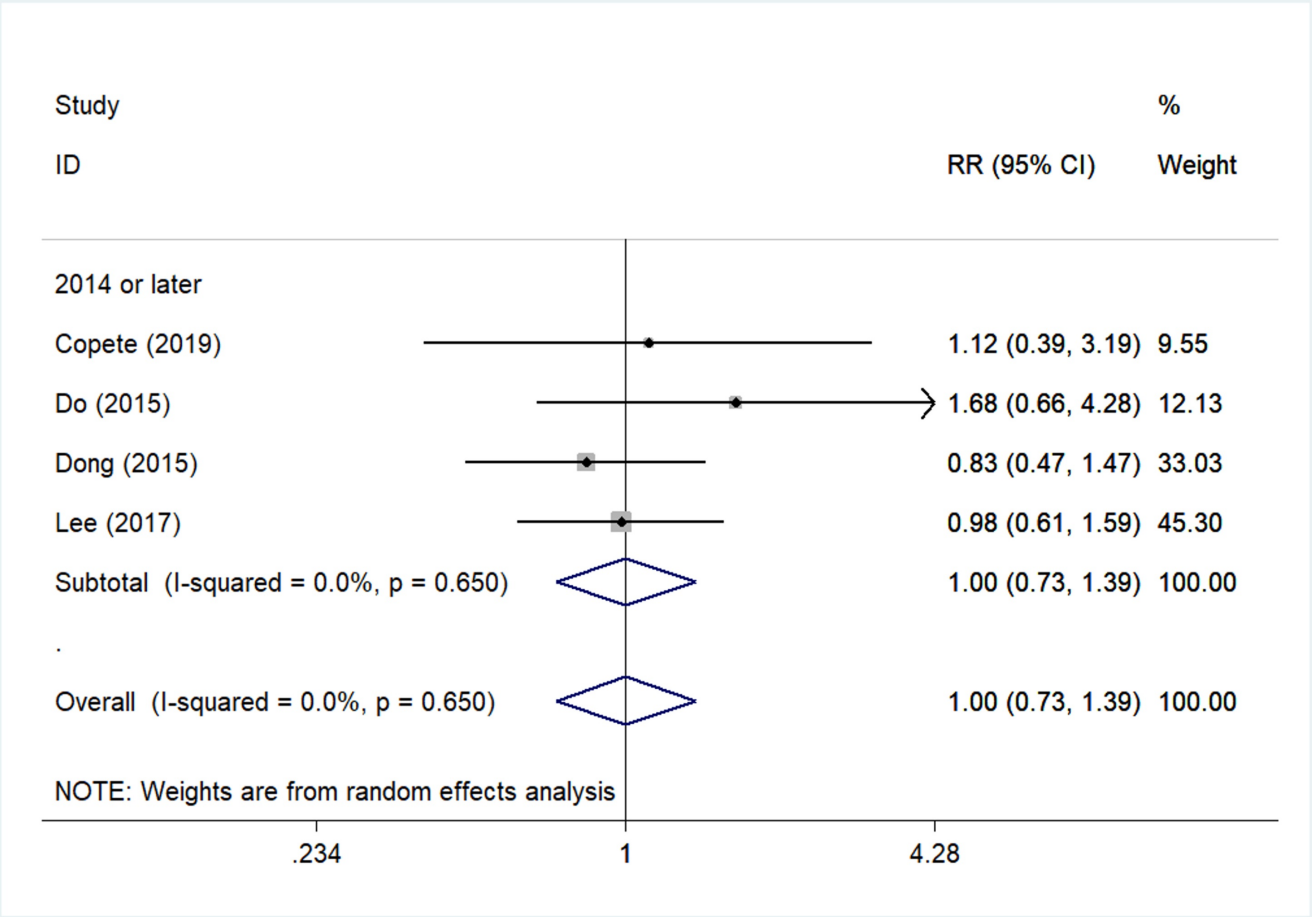
Forest plot describing the association between hypertension and risk for cystoid macular edema after uncomplicated cataract surgery. ID: identification; RR: relative risk; %: percent; p: p-value; I-squared: used for heterogeneity estimation Sub-analysis per publication year is presented. Bold cells denote statistically significant associations.

Hypertension: Four studies [24,27,32,39] analyzed the association between medical history of hypertension and PCME (Figure 3), leading to a null finding (pooled RR=1.00, 95%Cl: 0.73-1.39).

**Figure 4:**
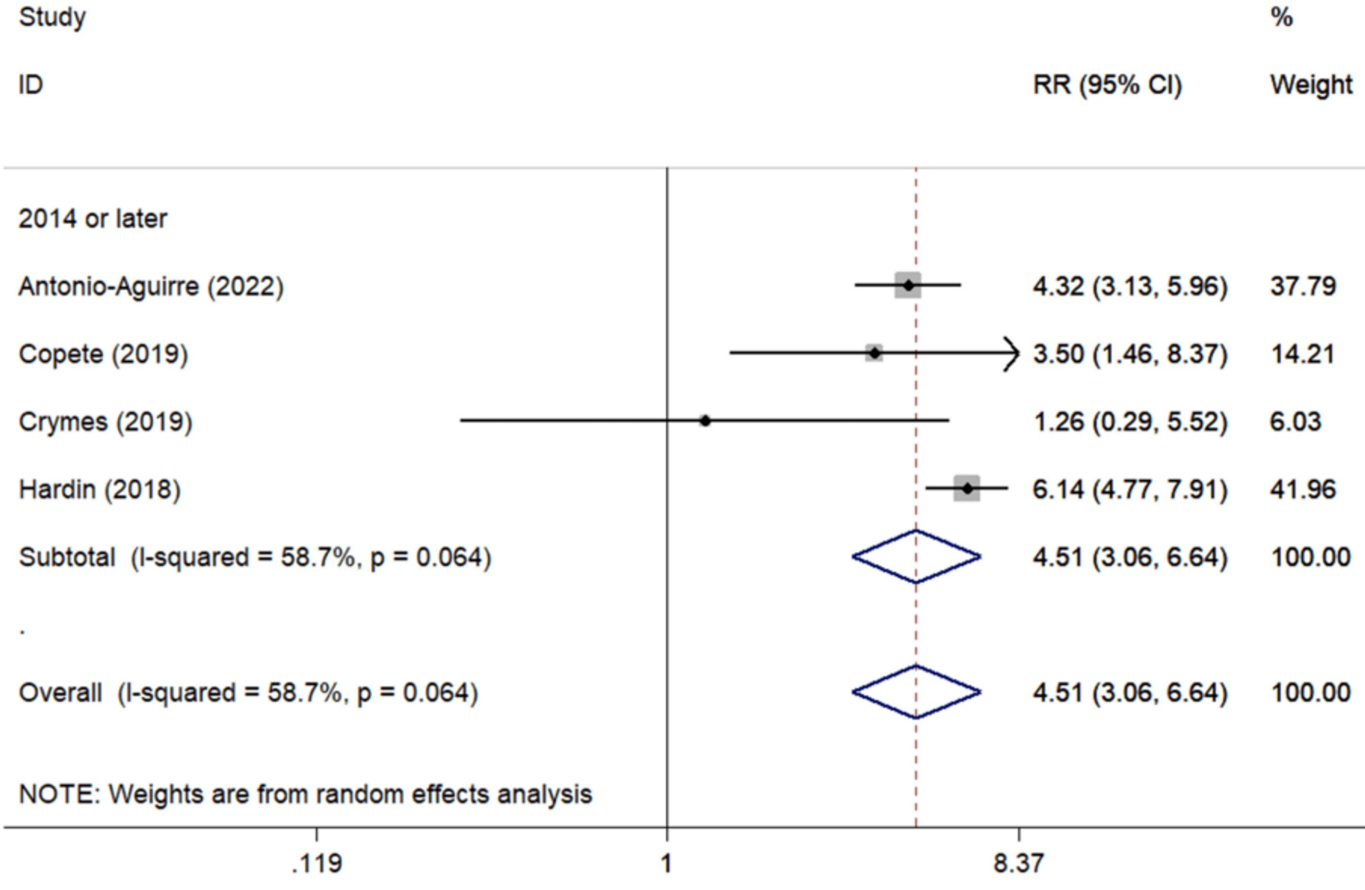
Forest plot describing the association between ERM and CME occurrence following uncomplicated cataract surgery. ID: identification; RR: relative risk; %: percent; p: p-value; I-squared: used for heterogeneity estimation; ERM: epiretinal membrane; CME: cystoid macular edema Sub-analysis per publication year is presented. Bold cells denote statistically significant associations.

Ophthalmological conditions: Concerning ophthalmological medical entities, five studies [25,34,39-41] examined the impact of DR upon PCME development, revealing an insignificant unfavorable effect upon PCME (pooled RR=3.87, 95%CI: 0.99-15.17). A significant unfavorable association was detected in the study derived from the past decade. A significant association between ERM history and PCME was noted (pooled RR=4.51, 95%Cl: 3.06-6.64, four studies (Figure 4)) [24,25,42,43]. The significant association was also observed in the subgroup analysis of the European population.

**Figure 5:**
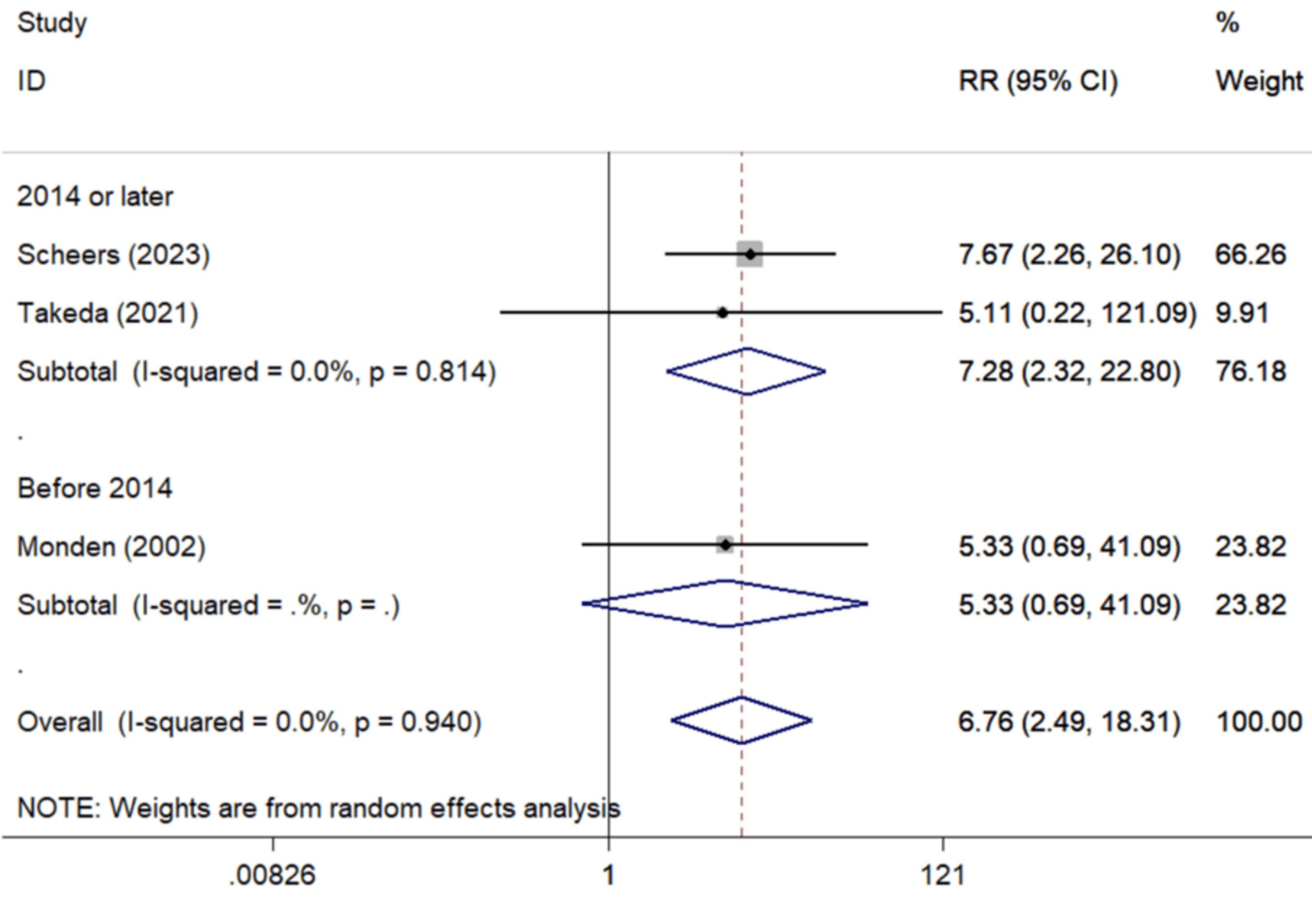
Forest plot describing the association between uveitis and CME occurrence following uncomplicated cataract surgery. ID: identification; RR: relative risk; %: percent; p: p-value; I-squared: used for heterogeneity estimation; CME: cystoid macular edema Sub-analysis per publication year is presented. Bold cells denote statistically significant associations.

**Figure 6:**
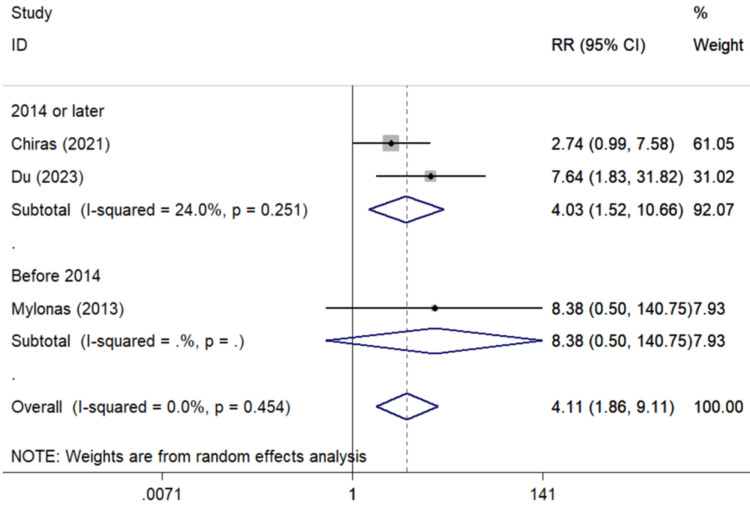
Forest plot describing the association between PPV and CME occurrence following uncomplicated cataract surgery. ID: identification; RR: relative risk; %: percent; p: p-value; I-squared: used for heterogeneity estimation, PPV: pars plana vitrectomy; CME: cystoid macular edema Sub-analyses per publication year are presented. Bold cells denote statistically significant associations.

Significant associations were also detected in the synthesis of three studies [40,44,45] correlating past uveitis and PCME occurrence (Figure 5) (pooled RR=6.76, 95%Cl: 2.49-18.31), and also of the three studies [46-48] associating previous PPV with PCME (pooled RR=4.11, 95%Cl: 1.86-9.11) (Figure 6).

**Figure 7:**
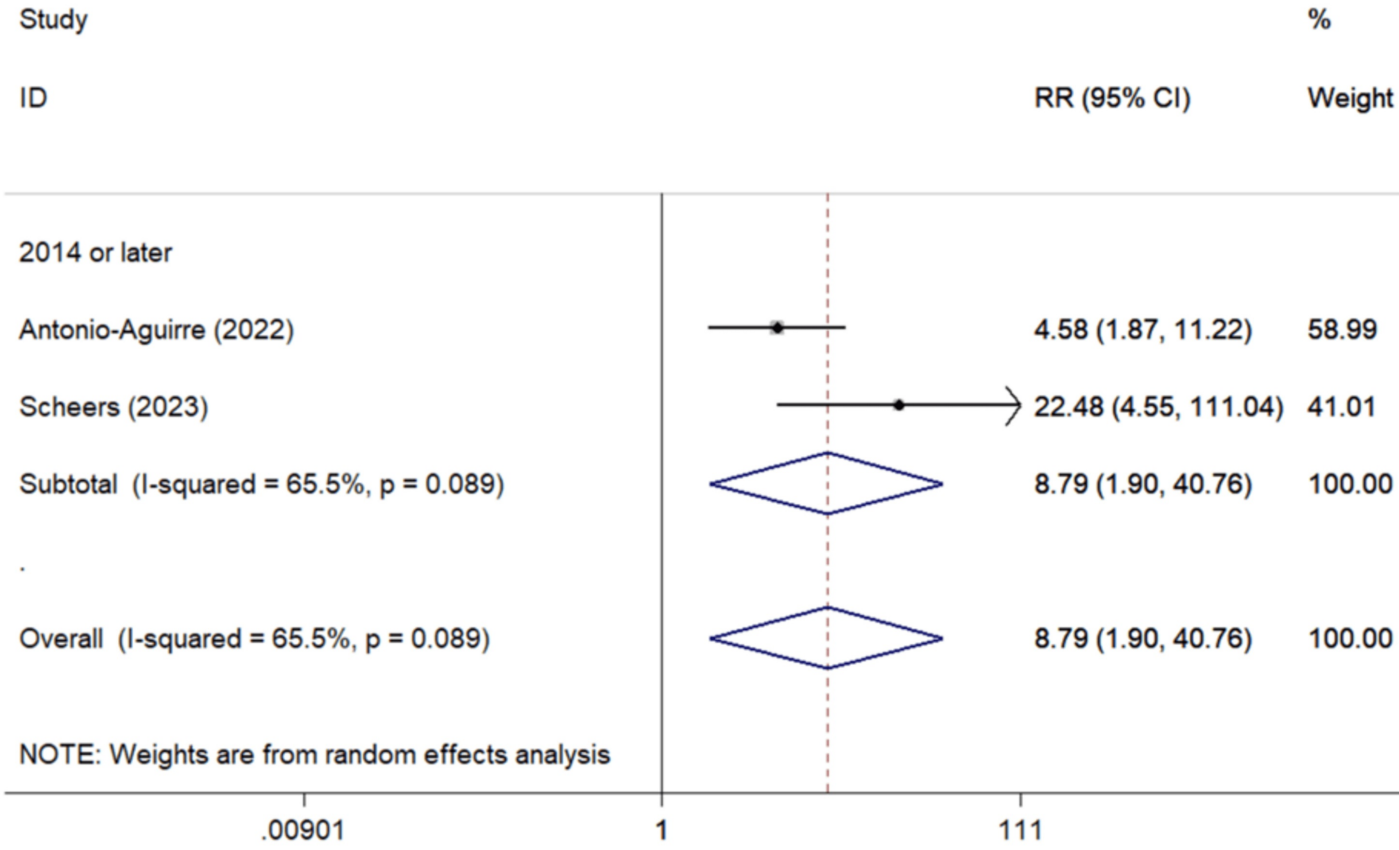
Forest plot describing the association between RVO and CME occurrence following uncomplicated cataract surgery. ID: identification; RR: relative risk; %: percent; p: p-value; I-squared: used for heterogeneity estimation; RVO: retinal vein occlusion; CME: cystoid macular edema Sub-analysis per publication year is presented.

The history of RVO was significantly associated with a higher danger of PCME incidence, as indicated by two studies (Figure 7) [40,42] (pooled RR=8.79, 95%Cl: 1.90-40.76). Based on evidence from two studies of the last decade involving European patients [24,40], a non-significant association between AMD and PCME (pooled RR=1.42, 95%Cl: 0.06-33.23) was observed; a protective effect of dry AMD, which did not reach statistical significance was observed in the analysis of two European derived studies (pooled RR=0.61, 95%Cl: 0.09-4.31, two studies) [40,49].

The association between past PVD and PCME (Table 3) did not reach significance in the meta-analysis of the two relevant studies (pooled RR=0.75, 95%Cl: 0.42-1.36) [24,50]. Surgical techniques: No significant associations were demonstrated either in the analysis on FLACS [51-54] (pooled RR=0.58, 95%Cl: 0.30-1.12) or SICS (pooled RR=1.61, 95%Cl: 0.54-4.79) [55,56].

**Table 3:** Results of the meta-analyses examining possible systemic and ophthalmological risk factors; subgroup analyses by study design and geographic region are presented. § number of studies; NC: not calculable; RR: relative risk; all studies presented data estimates after 2014 and therefore no subgroup analysis by publication year was presented; *all studies presented univariate estimates and therefore no subgroup analysis by adjustment was presented; **all studies presented multivariate estimates and therefore no subgroup analysis by adjustment was presented; †all studies were conducted in Europe; ‡all studies conducted in Asia

	n^§^	RR (95%CI)	Heterogeneity I^2^	P-value
Analysis on diabetes mellitus				
Overall analysis	19	2.87 (1.96-4.21)	80.3%	<0.001
Subgroups by publication year				
2014 or later	15	2.85 (1.89-4.31)	84%	<0.001
Before 2014	4	3.13 (1.11-8.83)	18%	0.301
Subgroups by adjustment				
Multivariate analysis	4	3.25 (1.64-6.44)	41.2%	0.164
Univariate analysis	15	2.76 (1.76-4.33)	83.5%	<0.001
Subgroups by geographic region				
Asia	8	3.06 (1.70-5.49)	69.2%	0.002
Australia	1	2.60 (0.87-7.78)	NC	
Europe	10	2.76 (1.55-4.90)	81.4%	<0.001
Analysis on hypertension §*				
Overall analysis	4	1.00 (0.73-1.39)	0.0%	0.650
Subgroups by geographic region				
Asia	3	0.99 (0.71-1.40)	0.0%	0.450
Europe	1	1.12 (0.39-3.19)	NC	
Analysis on diabetic retinopathy*				
Overall analysis	5	3.87 (0.99-15.17)	94.9%	<0.001
Subgroups by publication year				
2014 or later	4	3.37 (0.73-15.48)	96.2%	<0.001
Before 2014	1	8.00 (1.06-60.53)	NC	
Subgroups by geographic region				
Asia	3	2.15 (1.21-3.83)	18%	0.295
Europe	2	5.38 (0.48-60.57)	83%	0.015
Analysis on ERM§				
Overall analysis	4	4.51 (3.06-6.64)	58.7%	0.064
Subgroups by adjustment				
Multivariate analysis	2	4.21 (3.12-5.69)	0.0%	0.657
Univariate analysis	2	3.31 (0.73-15.08)	76.7%	0.038
Subgroups by geographic region				
Europe	2	5.45 (3.47-8.56)	32.2%	0.224
North America	2	2.90 (0.94-5.52)	60.8%	0.110
Analysis on uveitis*				
Overall analysis	3	6.76 (2.49-18.31)	0.0%	0.940
Subgroups by publication year				
2014 or later	2	7.28 (2.34-22.80)	0.0%	0.814
Before 2014	1	5.33 (0.69-41.09)	NC	
Subgroups by geographic region				
Asia	2	5.27 (0.95-29.29)	0.0%	0.982
Europe	1	7.67 (2.26-26.10)	NC	
Analysis on PPV*				
Overall analysis	3	4.11 (1.86-9.11)	0.0%	0.454
Subgroups by publication year				
2014 or later	2	4.03 (1.52-10.66)	24%	0.251
Before 2014	1	8.38 (0.50-140.75)	NC	
Subgroups by geographic region				
Europe	2	3.12 (1.20-8.11)	0.0%	0.465
North America	1	7.64 (1.83-31.82)	NC	
Analysis on RVO§**				
Overall analysis	2	8.79 (1.90-40.76)	65.5%	0.089
Subgroups by geographic region				
Europe	1	22.48 (4.55-111.04)	NC	
North America	1	4.58 (1.87-11.22)	NC	
Analysis on AMD §*†				
Overall analysis	2	1.42 (0.06-33.23)	87.5%	<0.005
Analysis on dry AMD §*†				
Overall analysis	2	0.61 (0.09-4.31)	0.0%	0.608
Analysis on PVD*				
Overall analysis	2	0.75 (0.42-1.36)	0.0%	0.611
Subgroups by publication year				
2014 or later	1	0.99 (0.29-3.34)	NC	
Before 2014	1	0.69 (0.35-1.36)	NC	
Subgroups by geographic region				
Asia	1	0.69 (0.35-1.36)	NC	
Europe	1	0.99 (0.29-3.34)	NC	
Analysis on FLACS §				
Overall analysis	4	0.58 (0.30-1.12)	15.6%	0.314
Subgroups by geographic region				
Australia	1	1.20 (0.44-3.30)	NC	
Europe	2	0.46 (0.14-1.50)	0.0%	0.566
North America	1	0.36 (0.14-0.90)	NC	
Analysis on SICS §*‡				
Overall analysis	2	1.61 (0.54-4.79)	0.0%	0.841

Meta-Regression Analysis

Table 4 presents the results of meta-regression analyses. The percentage of males and the mean age were not significantly associated with the cumulative incidence of PCME. The association between PCME and DM was not modified by any of the two examined variables.

**Table 4:** Meta-regression analysis concerning potential modifiers in the relation between cystoid macular edema and possible systemic and diabetes mellitus. §: number of studies; CI: confidence interval; %: percent; P: p-value

Variables	Category or increment	Cumulative incidence of PCME			Association between PCME and diabetes mellitus		
		n^§^	Coefficient (95%CI)	P	n^§^	Exponentiated coefficient (95%CI)	P
Percentage of males	10% increase	106	0.04 (-0.02 to +0.11)	0.183	18	-0.16 (-0.55 to +0.24)	0.419
Mean age of participants	10-year increase	103	-0.02 (-0.10 to +0.05)	0.553	15	-0.54 (-1.81 to +0.74)	0.380

Evaluation of the Quality of Studies and Risk of Bias

The quality was mainly compromised by the ascertainment of exposure and outcome in the case of cohort studies. This can be attributed to the fact that an objective finding, such as OCT, was not available pre-operatively and post-operatively for the majority of studies, as well as missing information on the completeness of follow-up. The quality was jeopardized in the aspect of non-adjusted estimates (comparability) in numerous articles.

Egger’s test detected significant publication bias in the analysis on overall cumulative PCME incidence (p<0.001). No significant publication bias was noted in the analysis examining the association between DM and PCME (p=0.100).

Discussion

This research comprised information from 143 discrete studies and is the first in this field consisting of this number of studies. It revealed that, overall, the incidence of PCME is 5% after uncomplicated cataract surgery. It is important to highlight that this meta-analysis showed that the cumulative incidence of PCME seems to be decreasing along with year progression, as indicated by the subgroup analysis by publication year, in which the cumulative incidence of PCME over the last decade is 5% (95%CI: 5%-6%). On the contrary, it is reported to be 7% (95%CI: 4%-10%) for surgeries performed before the last decade (Tables 1, 2). This may possibly be reflecting the introduction of new surgical techniques into clinical practice.

Synthesis of 37 studies revealed possible risk factors for PCME development, of which some proved to have a statistically significant result. DM was associated with a nearly threefold increase in the risk of PCME (Table 3). Diabetes is a chronic metabolic disease with inflammatory component, resulting in breakdown of BRB and consequent changes in the retinal vessel wall. Specifically, DR is characterized by pericyte loss, endothelial dysfunction, and thickening of the basement membrane, leading to vascular permeability and macular edema, as well as to retinal non-perfusion [159]. Cataract surgery causes post-operative inflammation, disrupting the BAB by surgical trauma, currently inclined since phacoemulsification was introduced in clinical practice, but not diminished [38]. The combination of endogenous and surgical co-existence seems to lead to PCME development [161]. Comparing our results with a previous meta-analysis published in 2015, the latter had not pointed to a significant association [162]. It is important to highlight that the impact of DM on PCME development has slightly decreasing during the last decade, as indicated by the subgroup analysis by publication year (2.85 vs 3.13 (Table 3, Figure 2)). This may be attributed to better screening, glycemic control, and new medications for DM.

Regarding the effect of the presence of ERM on PCME development, a statistically significant aggravating effect of this entity was spotted (Table 3, Figure 4). ERM is considered a medical entity in which the BRB integrity is often compromised. Cataract surgery, a known powerfully proinflammatory event [161], combined with ERM presence seems to increase the risk of PCME by inducing multiple insults to vascular permeability [162]. ERM can cause macular edema without cataract surgery, and CME itself is associated with generation of ERM [16]. European citizens with ERM have 1.879 times higher risk for PCME than North Americans (Table 3).

Commenting on the effect of uveitis on PCME, a highly significant association was detected, with patients with a history of uveitis having about 6.76 higher hazard for PCME (Table 3, Figure 5). A significant association was noted in the subgroup analysis concerning Europeans. Uveitic patients develop cataract at a younger age, and an adequate control of inflammation is necessary for surgery [163]. Furthermore, PCME development is common when uveitis co-morbidity is present. Postoperative aqueous flare may be increased even though intraocular inflammation quiescence is assured preoperatively and in spite of immunosuppression use [44]. A possible explanation is the BRB disturbance as a result of inflammatory mediators release in eyes more susceptible to inflammation [164].

PPV was proved to significantly increase the risk for PCME, by about four times, a result compatible with the subgroup analysis by publication year for studies performed during the last decade (4.03%). On the contrary, the risk of PCME was recorded eight times higher in studies carried out before 2014, in a statistically insignificant pattern (Table 3, Figure 6). This probably dictates the improvement of surgical technique in the past 10 years. In vitrectomized eyes, the macula may be potentially affected by the primary cause that led to the vitrectomy in the first place. In addition, post vitrectomy the retina is more exposed to the inflammatory agents released during cataract surgery, while the profile of the inflammatory agents’ flow may be altered as a result of vitreous loss, making it easier for them to approach the macula [46].

Concerning the impact of RVO history in PCME development, a significant harmful association was noted, with patients having up to nine times higher possibility for Irvine Gass (8.79%). It needs to be mentioned that Europeans with RVO history had 4.90 times higher risk for PCME, compared to North Americans, in a statistically significant pattern (Table 3, Figure 7).

Concerning the impact of hypertension history on PCME development, no statistical association was detected in our analysis. Lee et al. [27] and Do et al. [32] reported a harmful association between hypertension and PCME, but no statistical significance was reported in either of these studies (Table 3, Figure 3).

Concerning the impact of DR in PCME development, a harmful association was noted, even though the results didn’t reach statistical significance. The study performed before the last decade (Table 3) indicated a statistically significant negative association (8% vs 4% for more recent studies), probably reflecting the closer monitoring and further treatment choices for patients with DR during the last decade. Pathophysiologically, in diabetic eyes, especially those with DR, the post-surgery thickening of the macula is greater, compared to non-diabetic ones. The breakdown of the BRB, which is more intense in diabetic eyes, combined with cytokines including prostaglandin or vascular endothelial growth factor, which are released after cataract extraction, seems to play a role in PCME development after cataract surgery [165-167]. 

Only two studies examined the association between AMD and PCME development; their synthesis did not point to a significant association. The individual studies included were also controversial. In particular, Copete et al. [24] indicated that PCME development was unrelated to AMD presence, whereas Scheers et al. [40] detected a highly significant effect of AMD on PCME development, more frequently in the exudative subgroup. The effect of dry AMD upon PCME was also non-significant, in accordance with both synthesized studies [40,49]. 

It is worth to note that a common underlying mechanism for the previous-mentioned risk factors is the inflammatory cascade, which is triggered by cataract surgery. Therefore, it could be advisable to use prophylactic treatment for PCME in such patients [168]. No statistical significance was reached in the examination of PVD as a potential risk factor for PCME, in accordance with both included studies [24,50]. Regarding surgical techniques, a limited number of studies directly examined their effect upon PCME risk; their synthesis did not reach statistical significance, either for FLACS [51-54] or SICS [55,56].

In Van Nuffel et al. [54], the rate of PCME development was 3% lower in patients undergoing FLACS, but no statistical significance was proved. In Nithianandam et al. [53], a protective effect was also shown, and statistical significance was reached. In a more recent prospective randomized clinical trial of 104 patients by Conrad-Hengerer et al. [51], there were no significant differences in the occurrence of macular edema between laser-assisted cataract surgery and standard phacoemulsification. No large-scale study has compared clinical PCME across laser-assisted cataract surgery patients and manual cataract surgery patients. Levitz et al. [52] came in disagreement with the findings of the other studies, but no statistical significance was reached.

The present systematic review and meta-analysis presents some limitations, such as considerable heterogeneity between studies, which was detected in the overall analysis on the cumulative incidence of PCME. Differences in study design, geographical region, population size, follow-up duration and other factors are possible reasons for this heterogeneity; a series of subgroup analyses and meta-regression analyses was carried out in an attempt to detect its origins. Moreover, substantial publication bias was pointed out, indicating that effect of the presence of small studies, on the effect estimates [169]. As a result, a susceptibility to information and selection bias needs to be reported. Missing information on completeness of follow-up was a problem common in cohort studies. Finally, non-English studies were not included in this systematic review.

On the other hand, we need to note that the present work retains a majority of advantages. An updated search was made in PubMed and EMBASE. One hundred and forty-three studies were pooled in the quantitative synthesis of cumulative incidence. Furthermore, a considerable set of meaningful subgroup analyses was conducted on the available information, where the effects of endogenous risk factors frequently persisted.

## Conclusions

In conclusion, the results of this systematic review and meta-analysis estimate the cumulative incidence of the condition at 5%. This observation suggests that the cumulative incidence of PCME seems to be decreasing with year progression, a fact probably reflecting the closer monitoring and treatment of systemic and ocular comorbidities, as well as the new surgical techniques introduced in clinical practice. This research was the first in this field to provide statistically important information about endogenous risk factors and their association with the disease entity. Additional prospective cohort studies on risk factors, as well as large, randomized trials on newer surgical techniques, seem warranted.
